# Supplementary material for: Fully automated sequential immunofluorescence (seqIF) for hyperplex spatial proteomics
Source: Sci Rep. 2023 Oct 9;13:16994. doi: 10.1038/s41598-023-43435-w (PMC10562446; doi:10.1038/s41598-023-43435-w)
Supplement: Supplementary file 1 — Supplementary Information. [file 41598_2023_43435_MOESM1_ESM.pdf]

Supplementary Information

Qualitative Assessment of Repeatability and Reproducibility

To assess the repeatability and reproducibility of the developed method further, a 10-plex panel (CD68, FOXP3, CD8, PD-1, Ki-67, PD-L1, CK, CD4, CD20, CD3) on human tonsil FFPE tissue was run on 4 stainers of a COMET™ instrument in a single run (Figure S1a), as well as three times in a row on the same stainer (Figure S1b). Qualitative assessment of the results showed no visible variability on the obtained results.

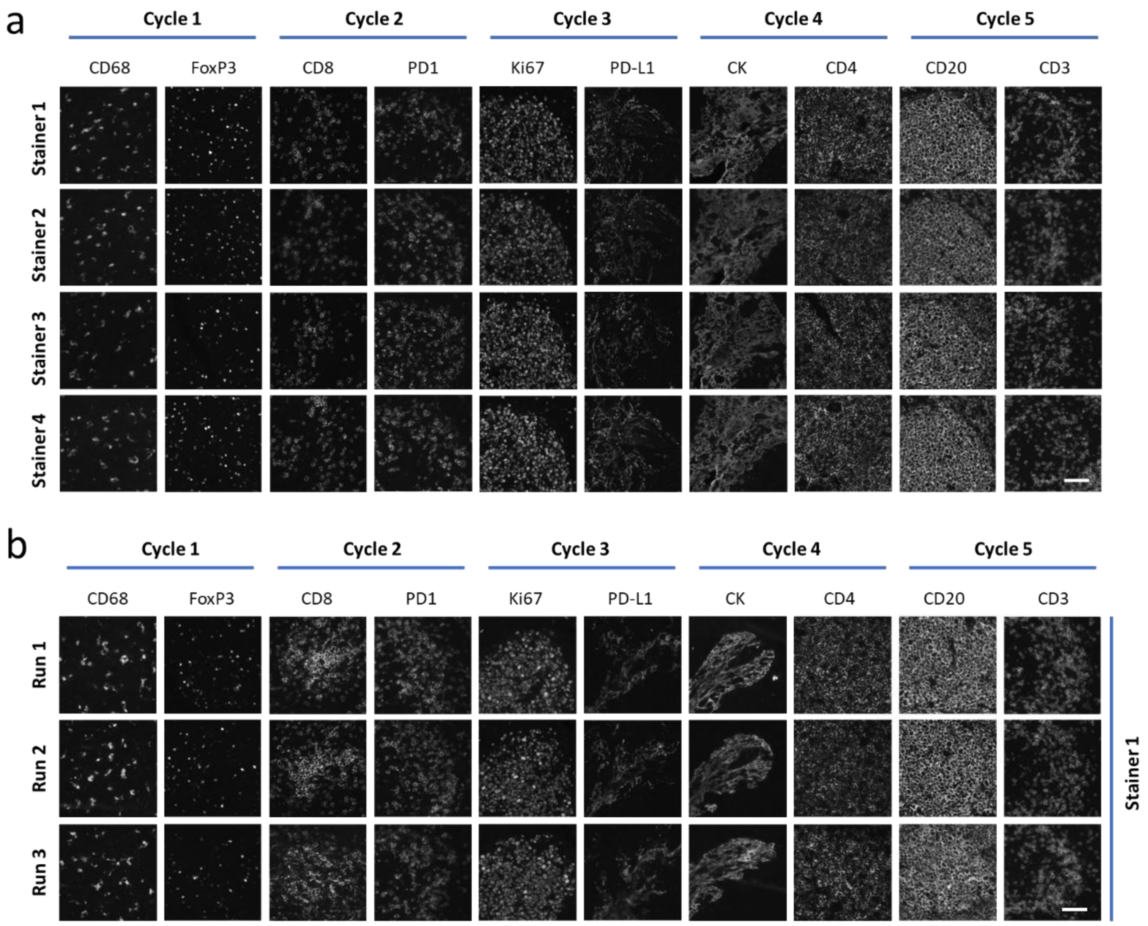

Figure S1: seqIF assays on COMET™ show high reproducibility between different stainers and independent runs.

(a) seqIF inter-stainer reproducibility study results on COMET™. A 10-plex panel protocol was run on the 4 stainer units of the same device and on the same day. Each image displays a representative ROI of a single marker. Scale bar: 50 µm.
(b) seqIF intra-stainer repeatability study results on COMET™, where consecutive runs of the same 10-plex protocol on multiple slides were executed on the same stainer unit of the same device. Scale bar: 50 µm.

User Workflow Overview for SeqIF

Figure S2 shows a broader view of the used seqIF workflow. It starts with sample pre-processing, which is performed in a PT Module. In parallel, device initialization, reagent preparation and loading of the reagents, protocols, and slide samples onto COMET™ is done. Once the slides are loaded in the instrument, the fully automated seqIF protocol starts. When needed, the method also allows the inclusion of pauses in the protocol to adjust imaging parameters in between cycles. This is a step that is typically needed during marker optimization or panel development, but to a lower extent in higher throughput experiments such as batch stainings since all parameters including imaging would be optimized beforehand. Considering that the developed method has a typical duration of 30 minutes per cycle, and 2 markers per cycle can be stained and imaged by using mixes of antibodies from different species, the process time for a typical 40-plex run is in the order of less than one day.

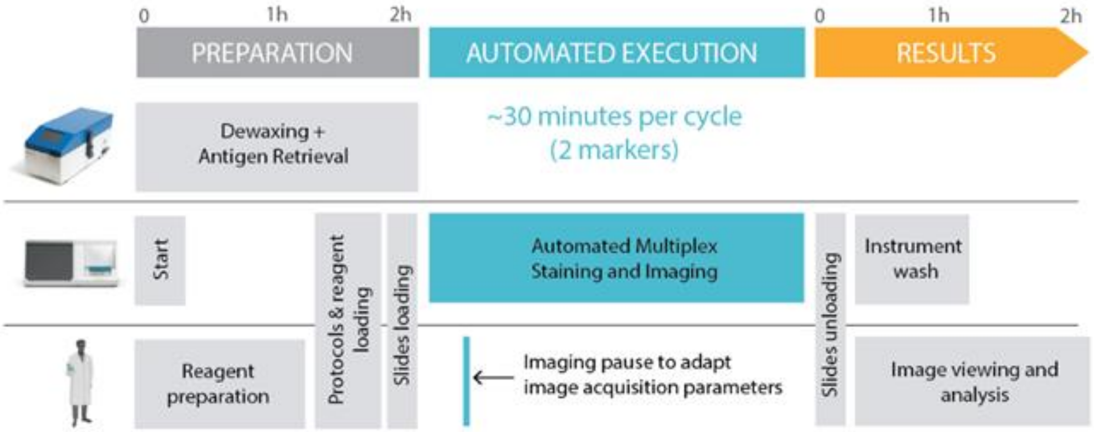

Figure S2: Overview of seqIF workflow, including timeframes for reagent and sample preparation, seqIF execution by automated platform and analysis of results.

Biomarker and Panel Optimization in seqIF

We have developed multiple seqIF marker and panel optimization strategies and characterization techniques to obtain results in the fastest time frame possible, and with the least amount of sample use. Figure S3a shows a representation of the developed approaches implemented in the form of different guided protocol templates available on COMET™. "Characterization Part 1" is a protocol that is used to assess the staining quality and elution efficiency of a new antibody prior to any optimization. It consists of a single staining cycle with imaging performed before and after the initial staining as well as after the elution step. An optional negative control step can be integrated after the elution. "Characterization Part 2" is designed to test different parameters on multiple cycles to determine optimal staining conditions. Images are acquired both after the staining and the elution steps. The user can set up to 20 cycles with different conditions. "Characterization Part 3" assesses the elution efficiency of a staining and its associated epitope stability over up to 20 cycles. SeqIF is the main protocol to run previously optimized multiplex staining protocols, for up to 20 cycles.

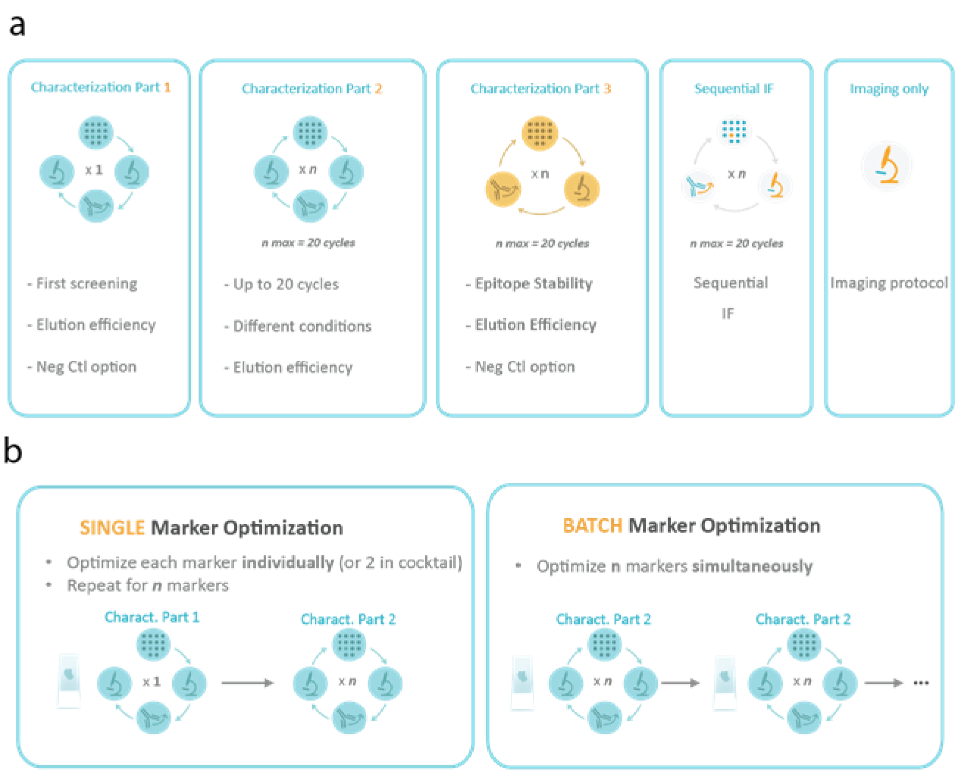

Figure S3: Guided optimization approaches on COMET™ for seqIF protocol development.

(a) Assisted optimization protocols developed for seqIF.

(b) Single and batch marker optimization modes used in panel development and optimization on COMET™. Neg Ctl: Negative Control - Tissue incubation with only secondary antibodies or with isotype controls of primary and secondary antibodies.

The two main optimization approaches for seqIF, single-marker and batch optimization modes, are shown in Figure S3b. During single marker optimization, up to 2 primary antibodies can be optimized for different conditions at each cycle using an initial Characterization Part 1 protocol, followed by Characterization Part 2 to test more conditions on the same tissue slide. If more than 2 markers require optimization, batch marker optimization becomes a useful approach. When batch optimization is preferred, multiple markers and conditions are tested in the same experiment using Characterization Part 2 protocols on consecutive tissue samples and optimize the desired number of markers simultaneously.

Figure S4 shows an example from the batch optimization approach where several markers are run in a relatively small panel, and parameters are adapted for each new run on a human lung adenocarcinoma FFPE sample on COMET™. The IHC references were provided by the tissue biobank. The 10-plex panel displayed was run as a 5-cycle protocol on a single tissue sample slide. Primary antibodies raised in different host species were mixed and detected by their corresponding secondary antibodies at each cycle. Using serial slides for each marker, a total of 4 runs were necessary for this panel optimization. Between each run, antibody concentration adjustment, primary antibody clone change, or the use of a blocking solution (blue arrows) were performed to reach the optimal final conditions in run #4.

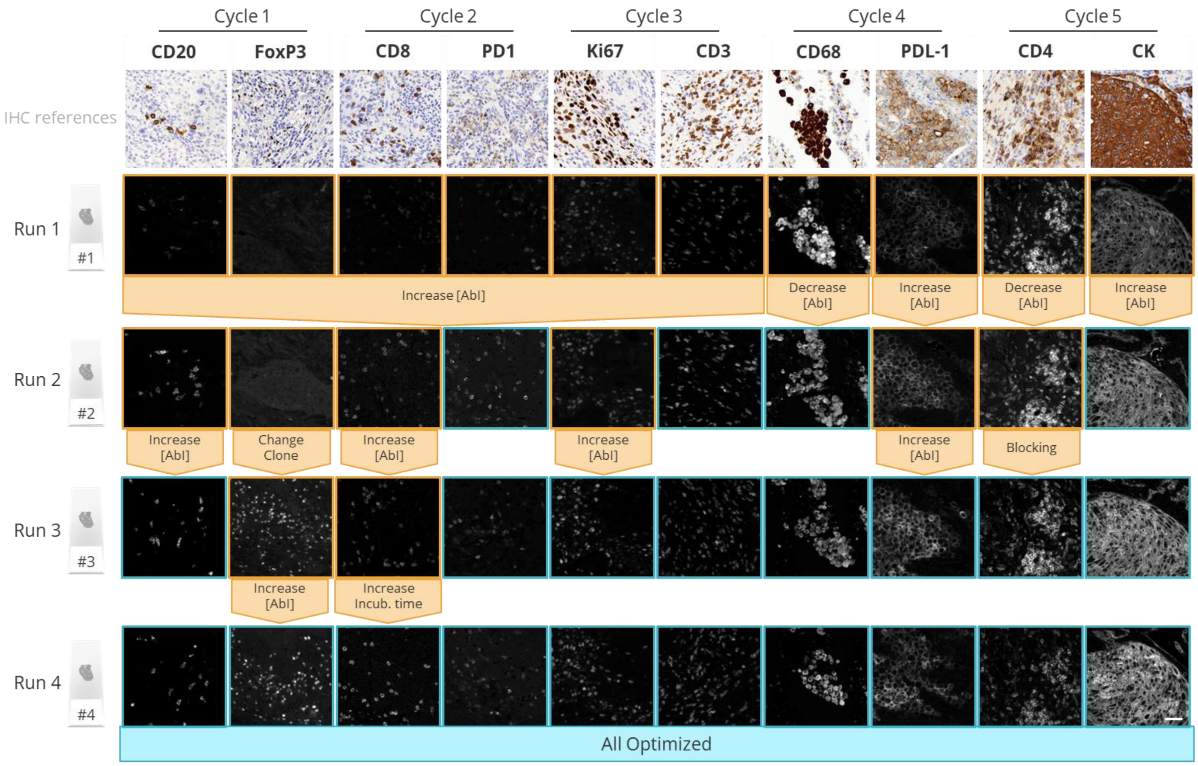

Figure S4: Batch marker optimization example. Optimization of a protocol performed on a human lung adenocarcinoma FFPE sample on COMET™, showing optimized results reached after 4 runs for a 10-plex panel.

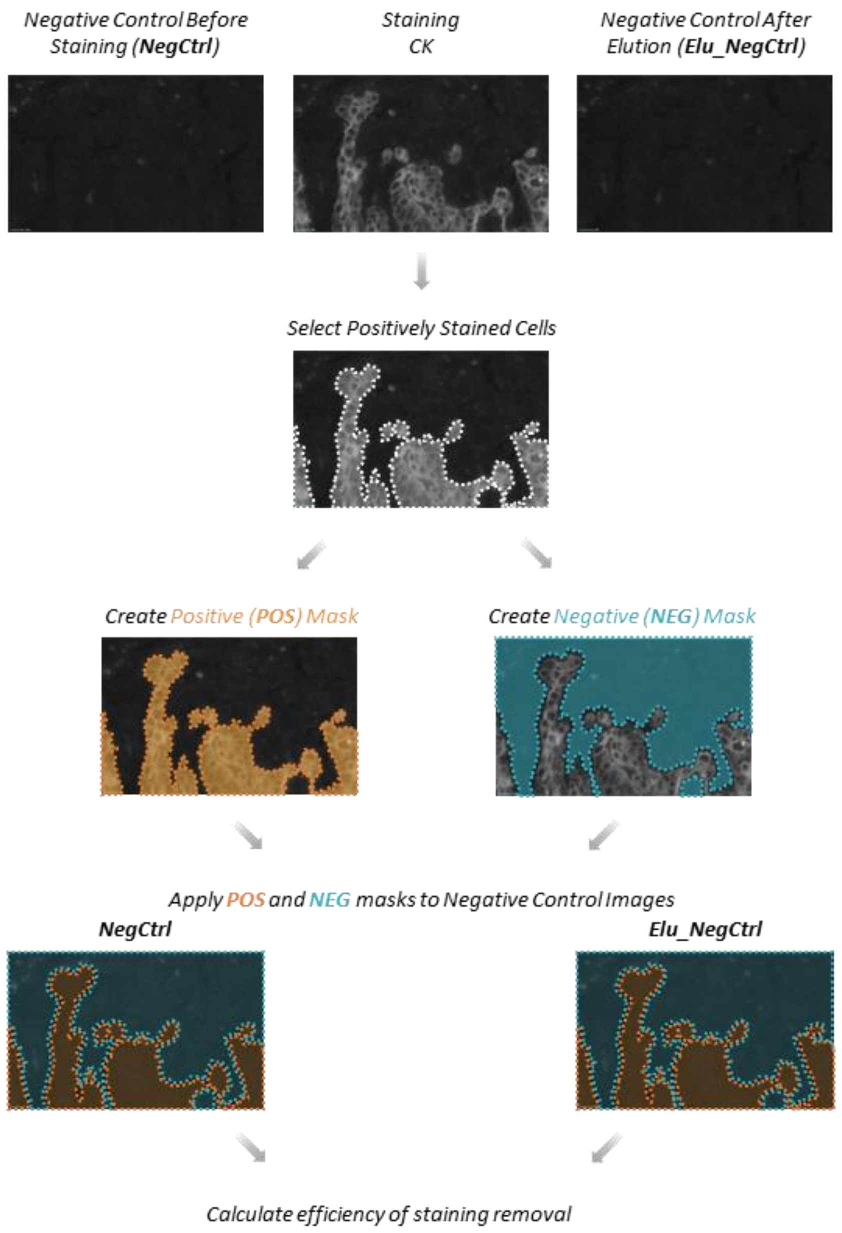

Figure S5: Demonstration of elution efficiency calculation workflow, where negative control images before staining and after elution are used in combination with the staining image to obtain the efficiency of staining removal.

NegCtrl: Negative Control - Tissue incubation with only secondary antibodies or with isotype controls of primary and secondary antibodies

Elu_NegCtrl: Negative Control after elution - Tissue incubation after elution of staining with secondary antibodies only.

Table S1: Primary antibody list and conditions for seqIF protocol.

Antigen	Antibody clone	Antibody Species	Supplier	Dilution range (1/)	Incubation time (minutes)
αSMA	1A4	Mouse	Cell Marque	200-400	4
BCL6	EP278	Rabbit	Cell Marque	50	4
CD107a	H4A3	Mouse	Biolegend	100	4
CD11b	EPR1344	Rabbit	Abcam	800	4
CD11c	EP157	Rabbit	Cell Marque	100	4
CD11c	EP1347Y	Rabbit	Abcam	1600	4
CD138	MI15	Mouse	Biolegend	100	4
CD14	EPR3653	Rabbit	Abcam	200-1000	4
CD15	CARB-3	Mouse	Dako	500	4
CD16	SP175	Rabbit	Cell Marque	100-400	4
CD163	MRQ-26	Mouse	Cell Marque	50	4
CD19	EPR5906	Rabbit	Abcam	200-400	4
CD20	L26	Mouse	Cell Marque	100-200	4-8
CD20	SP32	Rabbit	Thermo Scientific	100	4-8
CD21	EP3093	Rabbit	Cell Marque	400-1000	4
CD3	MRQ39	Rabbit	Cell Marque	200-1000	4-8
CD31	EPR3095	Rabbit	Abcam	400-800	4
CD34	EP88	Rabbit	CellMarque	400	4
CD38	SP149	Rabbit	Cell Marque	400-800	4
CD4	EPR6855	Rabbit	Abcam	100-400	4-8
CD45	PD7/26 + 2B11	Mouse	Dako	50	4
CD45	30-F11	Rat	Cell Signaling	1000	2
CD45RA	HI100	Mouse	BioLegend	1000-1600	4
CD45RO	UCHL1	Mouse	Dako	200	4
CD56	MRQ42	Rabbit	Cell Marque	100-600	4
CD68	KP1	Mouse	Thermo Scientific	50-100	4
CD8	4B11	Mouse	BioRad	50-100	4-8
CK	AE1/AE3	Mouse	Dako	50-150	4
E-Cadherin	36/E	Mouse	BD Bioscience	800-1000	4-8
F4/80	BM8.1	Rat	Cell Signaling	400	8
FOXP3	SP97	Rabbit	Thermo Scientific	25-50	4-8
GFAP	Polyclonal	Chicken	Abcam	1500	4
Granzyme B	EPR20129-217	Rabbit	Abcam	400-500	4

HLA-DR	TAL-1B5	Mouse	Santa Cruz	200-600	4
ICOS	EPR20560	Rabbit	Abcam	100-200	4
IDO-1	V1NC3IDO	Mouse	Invitrogen	400-500	4
Ki-67	MIB-1	Mouse	Dako	50-150	4
LAG-3	17B4	Mouse	Novus Bio	100-200	4
MYL9	EPR13012 (2)	Rabbit	Abcam	800	8
NaKATPase	H-3	Mouse	Santa Cruz	400	4
PD1	EPR4877(2)	Rabbit	Abcam	200-500	4-8
PD-L1	IHC411	Rabbit	GenomeMe	150	4
PD-L1	73-10	Rabbit	Abcam	500	4
Podoplanin	D2-40	Mouse	Agilent	40	8
S100	4C4.9	Mouse	Thermo Scientific	100-400	4
Tryptase	AA1	Mouse	Biolegend	1600	4
Vimentin	SP20	Rabbit	Cell Marque	100-200	4
Vimentin	SP20	Rabbit	Abcam	250	4
VISTA (B7-H5)	D1L2G	Rabbit	Cell Signaling	200	4
